# Evaluation of the tumor-targeting efficiency and intratumor heterogeneity of anticancer drugs using quantitative mass spectrometry imaging

**DOI:** 10.7150/thno.41763

**Published:** 2020-02-03

**Authors:** Jin Zhang, Qianqian Du, Xiaowei Song, Shanshan Gao, Xuechao Pang, Yan Li, Ruiping Zhang, Zeper Abliz, Jiuming He

**Affiliations:** 1 State Key Laboratory of Bioactive Substance and Function of Natural Medicines, Institute of Materia Medica, Chinese Academy of Medical Sciences and Peking Union Medical College, Beijing, 100050, China.; 2 Beijing Key Laboratory of New Drug Mechanisms and Pharmacological Evaluation Study, Institute of Materia Medica, Chinese Academy of Medical Sciences and Peking Union Medical College, Beijing, 100050, China.; 3 Center for Imaging and Systems Biology, Minzu University of China, Beijing, 100081, China.

**Keywords:** quantitative mass spectrometry imaging, whole-body animal, paclitaxel, tumor-targeting efficiency, intratumor heterogeneity

## Abstract

The development of improved or targeted drugs that discriminate between normal and tumor tissues is the key therapeutic issue in cancer research. However, the development of an analytical method with a high accuracy and sensitivity to achieve quantitative assessment of the tumor targeting of anticancer drugs and even intratumor heterogeneous distribution of these drugs at the early stages of drug research and development is a major challenge. Mass spectrometry imaging is a label-free molecular imaging technique that provides spatial-temporal information on the distribution of drugs and metabolites in organisms, and its application in the field of pharmaceutical development is rapidly increasing.

**Methods**: The study presented here accurately quantified the distribution of paclitaxel (PTX) and its prodrug (PTX-R) in whole-body animal sections based on the virtual calibration quantitative mass spectrometry imaging (VC-QMSI) method, which is label-free and does not require internal standards, and then applied this technique to evaluate the tumor targeting efficiency in three treatment groups—the PTX-injection treatment group, PTX-liposome treatment group and PTX-R treatment group—in nude mice bearing subcutaneous A549 xenograft tumors.

**Results**: These results indicated that PTX was widely distributed in multiple organs throughout the dosed body in the PTX-injection group and the PTX-liposome group. Notably, in the PTX-R group, both the prodrug and metabolized PTX were mainly distributed in the tumor tissue, and this group showed a significant difference compared with the PTX-liposome group, the relative targeting efficiency of PTX-R group was increased approximately 50-fold, leading to substantially decreased systemic toxicities. In addition, PTX-R showed a significant and specific accumulation in the poorly differentiated intratumor area and necrotic area.

**Conclusion**: This method was demonstrated to be a reliable, feasible and easy-to-implement strategy to quantitatively map the absorption, distribution, metabolism and excretion (ADME) of a drug in the whole-body and tissue microregions and could therefore evaluate the tumor-targeting efficiency of anticancer drugs to predict drug efficacy and safety and provide key insights into drug disposition and mechanisms of action and resistance. Thus, this strategy could significantly facilitate the design and optimization of drugs at the early stage of drug research and development.

## Introduction

Due to the rapid population growth and increase in the ageing population worldwide, cancer incidence and mortality have also significantly increased and can negatively affect human health and quality of life 1. Antineoplastic agents, the most commonly used malignant tumor treatment among the available methods, have gradually evolved from relatively nonspecific cytotoxic agents to selective, mechanism-based therapeutics and emerging immunomodulators 2. However, these drugs are seriously limited by significant off-target toxicities 3, poor tumor permeation 4 and frequently acquired resistance 5. To overcome these shortcomings, researchers have developed various targeting strategies that may enhance the tumor-discrimination capabilities to achieve the objective of killing tumors while avoiding serious undesirable side effects. For example, the tumor receptor-based target strategy 6 involves attachment of specific tumor recognition elements to anticancer compounds, such as antibodies 7, amino acids 8, folic acid 9 and nucleolin aptamers 10, which are overexpressed (or their receptors are overexpressed) or specifically expressed at tumor sites. In addition, excellent tumor-targeting drug delivery systems (DDSs) 11, 12 such as micelles, liposomes and intelligent nanoassemblies, have emerged as a pivotal breakthrough in treating solid tumors, ensuring that sufficient levels of drugs accumulate at the tumor site and infiltrate cancerous tissue to attack tumors with a high specificity.

Nevertheless, regardless of the type of anticancer drug used, the distribution of drugs or candidates in the target and non-target regions is the material basis of drug efficacy and potential toxicity. In other words, the drugs can exert their positive therapeutic effects only when they are present in the vicinity of the tumor at sufficient concentrations for an appropriate period of time; in contrast, undesired accumulation of a drug candidate or its metabolites in healthy organs will generate corresponding toxicity 13, 14. With our deep understanding of intratumor heterogeneity, an individual solid tumor tissue involves not only billions of cancer cells, which evolve into multiple tumor subclones with different degrees of drug resistance under the drive of genetic or epigenetic alterations and evolutionary selection^15^-^17^, but also considerable and codeveloped stromal cells and a large variety of extracellular matrix components, which could form a firm barrier preventing drug access to cancer cells and leading to moderate therapeutic efficacy 18-20. Further in-depth and detailed research on the intratumor distribution of anticancer drugs with heterogeneous characteristics will help elucidate the mechanisms of drug efficacy and resistance, thereby promoting the development of clinically useful formulations to prevent cancer progression and metastasis. Therefore, determination of the distribution of antitumor drugs within the native biological system and intratumoral microregions in advance is extremely important in the early stages of drug development and research and will further contribute to a detailed understanding of drug metabolism and pharmacokinetics (DMPK).

Various analytical methods and technologies have been developed to obtain information about the concentrations of drugs and their metabolites in different tissues. Historically, liquid chromatography coupled to mass spectrometry (LC-MS) has been widely used to identify drug distribution in the pharmaceutical industry 21. However, spatial localization information is lost with this strategy, and the obtained results are also average due to the need for time-consuming preparation processes, in which compounds and their respective metabolites are extracted from tissue homogenates. Spatially resolved biodistribution analysis at the whole-body animals level has generally been performed using molecular imaging technology, including whole-body autoradiography (WBA) 22, positron emission tomography 23, and fluorescence imaging 24, 25. These analyses mostly rely on radiolabelled tracers or report probes and thus do not distinguish between the parent drug and its potential metabolites that have retained the tag, leading to unreliable and inaccurate assessments of the drug tumor-targeting efficiency. Additionally, since few analytes throughout the section can be detected based on the presence of a radiolabel only, specific information for each chemical specie associated with these techniques is lacking, which limits the chemical information that can be obtained 26, 27.

Among the multiple techniques available, mass spectrometry imaging (MSI) technology appears to be a promising approach for simultaneously visualizing and identifying the spatial-temporal distribution of numerous different molecular analytes present within biological samples, including the drug, its metabolites, and functional endogenous components, without the need for affinity tags or expression markers in a single experiment. MSI technology can be divided into different categories based on recently developed multiple ionization methods. In particular, airflow-assisted desorption electrospray ionization (AFADESI)-MSI is a high sensitive, wide coverage, and high chemical specific molecular imaging technique that was developed by our group 14, 28 and mainly applied to pharmacological target screening 29, assessment of pharmaceutical agents distribution 30, and diagnostic and prognostic marker discovery 14. However, the drug's signal intensities obtained by this novel technique demonstrate its inability to objectively reflect the absolute content of the drug in different tissues owing to sample heterogeneity, ion suppression, analyte extraction efficiency and ionization efficiency; thus, accurate quantitation by MSI remains a challenge 31. Generally, the most recommended strategy used for correcting for ion signal variability is incorporation of a stable-isotope-labelled internal standard (SILIS) or a close analogue as an internal standard in quantitative MSI (QMSI) 32-34. This type of quantitative analysis cannot be easily conducted, especially with many potentially therapeutic agents screenings at the early stage of drug discovery, because the additional synthesis of SILISs is both costly and time-consuming.

In this study, we used a potentially antitumor prodrug (PTX-R,**Figure S1A**) of paclitaxel (PTX, **Figure S1B**) as the researching case, and achieved accurate pixel-to-pixel quantitation for the distribution of PTX and PTX-R in whole-body animal sections based on a label-free and internal standard-free virtual calibration quantitative MSI (VC-QMSI) method established recently by our group 35. AFADESI-MSI was used to simultaneously acquire the MS signal corresponding to drugs, their metabolites and the endogenous metabolites, and then, the endogenous metabolites were used as native internal standards. A neural network model was established between the response of the endogenous metabolites and the relative matrix effect of the drugs through machine learning. In addition, the actual drug content and distribution in an unknown dosed sample was corrected based on the relative matrix effect predicted by the developed model. This quantitative method was then applied to evaluate the tumor targeting efficiency of three treatment groups—the PTX-injection treatment group, PTX-liposome treatment group and PTX-R treatment group—of nude mice bearing subcutaneous A549 xenograft tumors.

## Materials and Methods

### Chemicals and Materials

LC-MS grade acetonitrile, methanol and isopropanol were purchased from Thermo Fisher Scientific (Massachusetts, USA). Purified water was obtained from Wahaha (Hangzhou, China). The drug standard PTX was purchased from Zhenzhun Biotechnologies Co., Ltd. (Shanghai, China). The modified PTX (PTX-R) was provided by BioDuro Shanghai Co., Ltd. PTX-R was prepared as 15% (w/v) hydroxypropyl-β-cyclodextrin complexes. The PTX injection and PTX liposomes were purchased from the North China Pharmaceutical Group Co., Ltd., and Yangtze River Pharmaceutical Group, respectively. The active pharmaceutical ingredient (API) of PTX liposomes is paclitaxel, and the excipients are lecithin, cholesterol, threonine, and glucose. Chemical structures and corresponding high-resolution mass spectra of PTX and PTX-R were shown in **Figure S1**.

### Animal Study

These studies were conducted in accordance with the Guide for the Care and Use of Laboratory Animals 36 and were approved by the Animal Care & Welfare Committee, Institute of Materia Medica, Chinese Academy of Medical Sciences and Peking Union Medical College (Beijing, China). Male 8-week-old Balb/C mice (purchased from the National Institutes for Food and Drug Control, Beijing) were selected as the test animals. Balb/C mice were inoculated subcutaneously with A549 lung cancer cell suspension mixed with Matrigel to establish the xenograft tumor model. Tumors were observed 2 weeks after inoculation. The tumor-bearing nude mice were randomly divided into three treatment groups that were administered 37.5 mg/kg PTX injection, 37.5 mg/kg PTX liposome, 43.2 mg/kg PTX-R (equal mole dosage with PTX), respectively, by intravenous route (i.v.) via the tail vein. The mice were killed by CO_2_ gas at 0.5, 3, 11, 24 hours post-dose administration (n = 3 for each time point). Tumors were collected and intact whole-body were kept, and stored at -80 °C for following QMSI analysis. The detailed schedule for the treatment scheme in the xenograft tumor model is shown**Figure S2**.

### Whole-body Animal Section and Tumor Tissue Section Preparation

Individual mouse carcass was embedded into a regular block with 3% (w/v, g/100 mL) aqueous carboxymethyl cellulose. The corresponding removed tumor tissue was embedded in OCT. The 25-μm-thick whole-body animal sagittal cryo-sections and tumor tissue sections with the same thickness were cut using a Leica CM3600 cryostat microtome and a Leica CM1860 cryostat microtome (Leica Microsystem Ltd., Germany) at -20 °C, respectively. The tissue sections were always stored in a closed slice box at -80 °C until the subsequent analysis. The frozen slices were first dried in a vacuum container for one hour and then transferred to room temperature for another hour prior to AFADESI-MSI analysis. Serial tumor tissue sections were fixed with 4% paraformaldehyde and subsequently stained using H&E for pathological examination.

### Mimetic Tissue Model Preparation

The two groups of drug-spiked mimetic tissue models were prepared to generate a neural network model to predict the relative matrix effect of each pixel and quantitative calibration curve. Standard curve model was constructed separately using the following steps. As shown in **Figure S3**, a series of drug reference standards were spiked into the diluted tissue homogenates of different organs, 5 μL of the mixture was drawn into the well of a self-custom mold, and a holding time of 5 mins was used to allow the sample to dry before each dried point was covered using the tissue sections of the corresponding organs. Finally, standard curves with different concentrations of 0.89, 1.78, 4.45, 8.9, 13.35, 17.8, 35.6, and 71.2 pmol/mm^2^ for PTX and 0.079, 0.158, 0.79, 1.58, 3.95, 7.9, 15.8, and 31.6 pmol/mm^2^ for PTX-R were constructed. Another mimetic tissue model for predicting the relative matrix effect factors was constructed in the same way as above except the same concentration was used at each point.

### AFADESI-MSI Analysis

MSI data acquisition was performed using a lab-built AFADESI-MSI platform, which consists of an AFADESI ambient ion source and a Q-Orbitrap mass spectrometer (Q-Exactive, Thermo Fisher Scientific, USA) 37, 38. In the MSI experiments, the whole-body tissue sections were fixed on an electrical moving stage that moves at a 350 μm horizontal speed and a 500 μm vertical step size (MTS225, Beijing Optical Instrument Factory, Beijing, China), and each section was continuously scanned at line-by-line. A mixed solution of acetonitrile: water (5:5, v/v) was used as the optimized spray solvent with a flow rate set to 10 μL/min. The analysis was carried out using an alternative scan mode combining full MS with t-SIM in positive ion mode, which simultaneously obtained abundant information on drugs, drug metabolites and endogenous compounds. A detailed parameter setting is shown in **Table S1**. The acquisition rate of the mass spectrometer is 2.4 scan/s under these parameters. The spatial resolution of AFADESI‐MSI system was approximately 150 μm by estimation in this study.

### VC-QMSI Data Processing

The data processing of QMSI was performed with MATLAB 2018a (The MathWorks, USA) using self-written MATLAB scripts 35. The raw data collected by Q-Exactive were first transformed into a .cdf format file using Xcalibur (Thermo Fisher Scientific, USA) and imported into MATLAB 2018a to form a three-dimensional cell, which read and saved the information of the spatial location and intensity of all detected ions, for further subsequent data filtering, compression, dimensionality reduction, feature extraction, image reconstruction, etc. First, we selected the endogenous metabolites as input features that are highly correlated with drug response intensity (Pearson correlation coefficient >0.4), calculated the relative intensity of the drug in different types of reference tissues (the relative matrix factors) as the output target according to drug-spiked mimetic tissue models of the same drug concentration in different types of reference tissues, and then chose Levenberg-Marquardt as the training algorithm in the Neural Network Toolbox to develop the regression models. The t-distributed stochastic neighbour embedding (t-SNE) and k-means clustering (k-means) were used to perform the spatial segmentation analysis in an open source toolbox in MATLAB.

### Statistical Analysis of VC-QMSI Data

Statistical analysis was performed using GraphPad Prism 6 (San Diego, CA, USA). Paired and unpaired two-tailed Student's *t*-tests were used for comparing difference between different groups or different tissues and P values were plotted (*, significantly different between two groups at p < 0.05; **, p < 0.01 and ***, p < 0.001). Results are presented as mean ± SEM (standard error of the mean). For optimization of spray solvents and comparison of drug contents in different organs and treatment groups, n = number of replicates from 3 mice.

## Results and Discussion

### VC-QMSI Strategy Modelling Based on an Artificial Neural Network (ANN)

**Figure 1:**
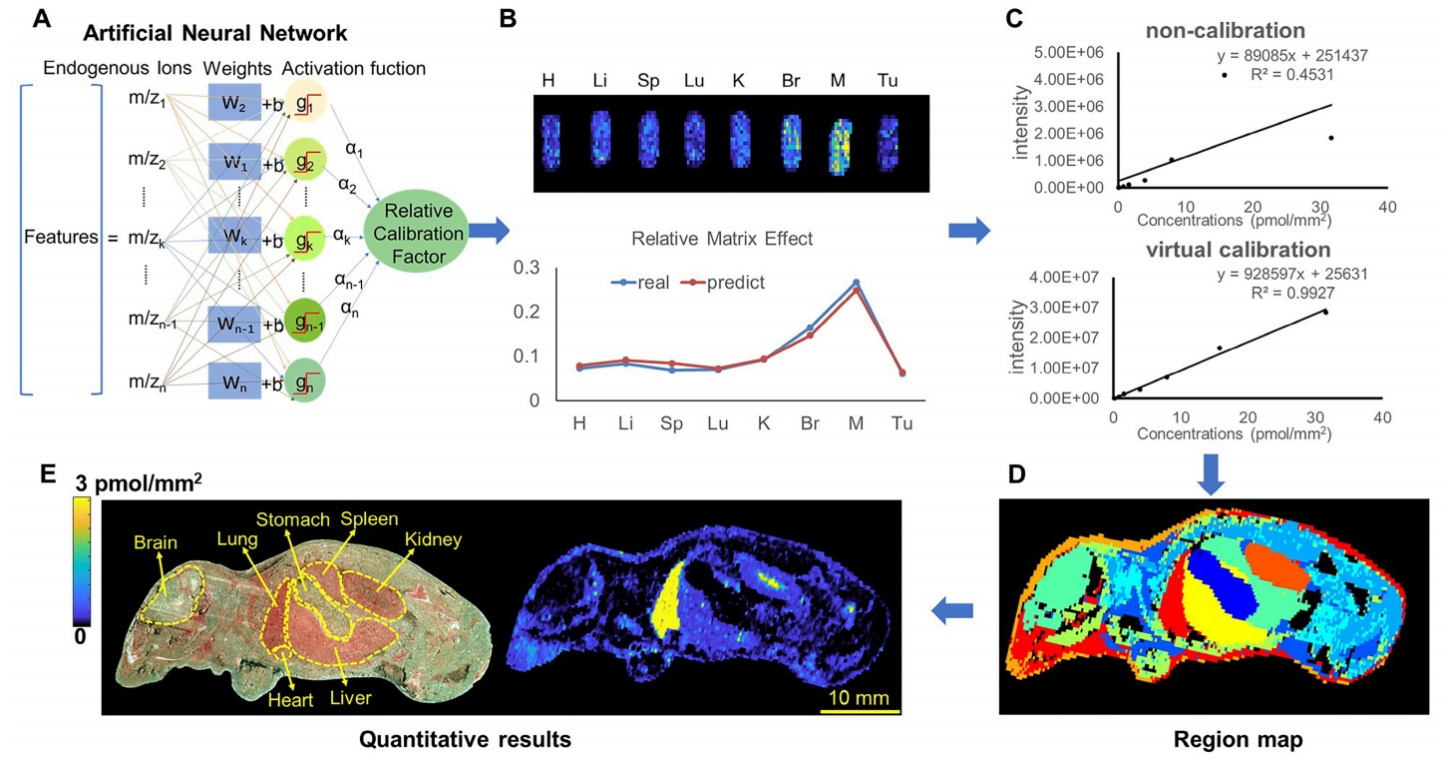
Schematic diagram depicting the process by which the VC-QMSI strategy accurately maps antitumor drugs in whole-body animal tissues. (A) Schematic illustration of the machine learning method to predict the relative calibration factor based on the endogenous metabolites. (B) The imaging of relative calibration factors of different organs and the comparison between predicted and true values of the relative calibration factor. Abbreviations: H, heart; Li, liver; Sp, spleen; Lu, lung; K, kidney; Br, brain; M, muscle; Tu, tumor. (C) The non-calibration and virtual calibration standard curves constructed with the drug amount versus the non-calibrated and calibrated drug ion intensities, respectively. (D) The image of whole-body sample segmentation by automatic pixel labelling using K-means and t-SNE clustering analysis. (E) The final result of drug quantitative visualization in each organ and the optical image of the whole-body animal sample.

By using different types of mimetic tissue references with the same amount of drug as the modelling sample, as shown in **Figure 1A**, we established a predictable ANN model between the relative intensity of the drug (relative calibration factors, RCF) and the ion intensities of the screened endogenous metabolites. The pixels from two-dimensional data matrix were randomly divided into training set, validation set, and test set according to the ratio (7.0: 1.5: 1.5) for model construction, in-group and out-group verification, the results were shown in **Figure S4**, indicating that the ANN model achieved a better training accuracy and was not overfitted. Notably, the predicted RCF of each type of organ or tissue demonstrated an ideal consistent with the actual value (**Figure 1B**), which was applied to calibrate the ion intensity variation of the drug due to the matrix effect in a pixel-by-pixel manner in quantitative standard curve samples and dosed tissue samples using the formula:

Intensity_cal_=Intensity/RCF_predicted_

The correlation coefficient of the established standard curve increased from 0.45 to 0.99 after virtual calibration (**Figure 1C**). The VC-QMSI strategy modelling results for PTX are shown in **Figure S5**. Next, the calibrated standard curve was utilized to determine the absolute concentrations of drugs within dosed whole-body animal sections. In addition, the VC-QMSI strategy enables the automatic recognition of spatial regions based on the characteristics of abundant endogenous metabolites containing the MS image pixel point through machine learning instead of manually selecting the region of interest (ROI) under the guidance of optical or H&E imaging **(Figure 1D)**. This approach will help improve the accuracy and intelligence of QMSI. Finally, our results demonstrated that VC-QMSI can be successfully used for spatially resolved quantitative analysis of drug candidate distribution in complicated whole-body animal samples **(Figure 1E)**.

### Optimization of a spray solvent for PTX and PTX-R

**Figure 2:**
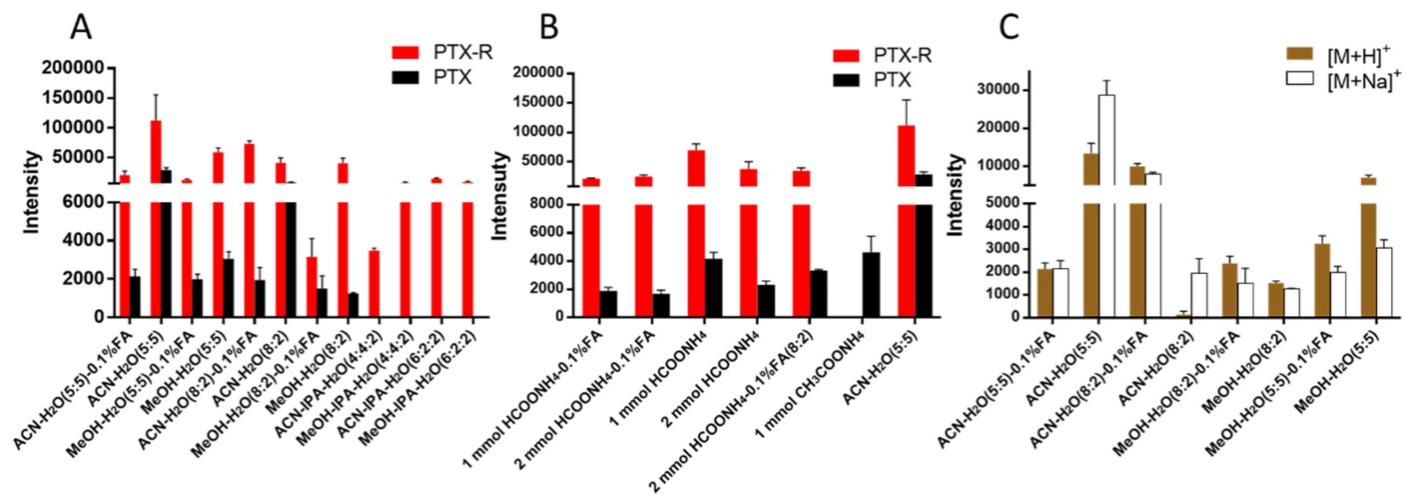
The statistical analysis of the ion intensities of PTX and PTX-R in equivalent-amount drug-spiked mimetic tissue models under different spray solvents. (A) The screening results for the composition of the organic and aqueous phases of the spray solvent. (B) The effect of MS-tolerant volatile salt addition on sensitivity based on the optimal spray solvent. (C) Quantitative ion selection of PTX under different spray solvents.

**Figure 3:**
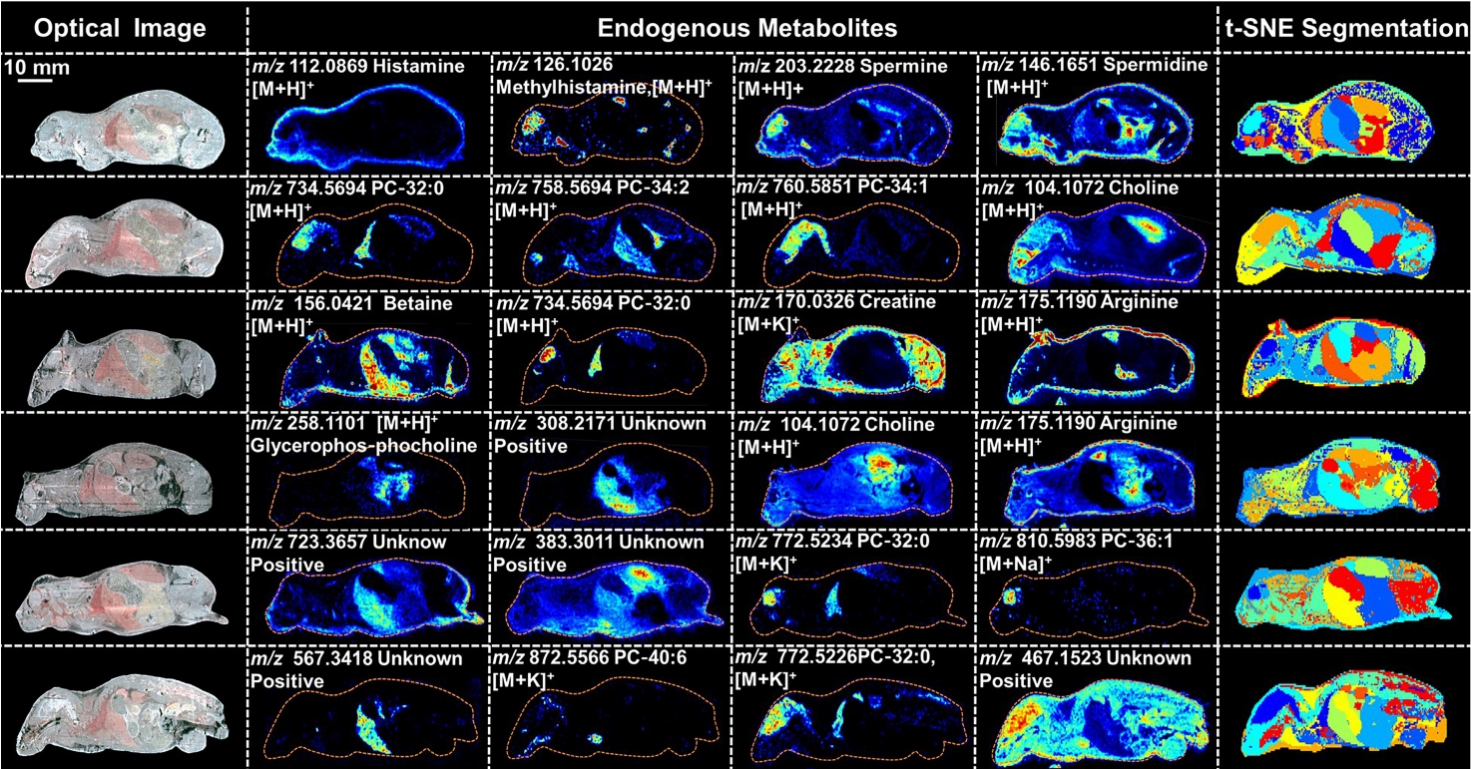
Optical images, MS images of representative tissue-specific metabolites obtained by AFADESI-MSI under optimized conditions and t-SNE spatial segmentation of physiological regions based on metabolite profiling in highly complicated whole-body animal sections.

PTX is a hydrophobic compound with a low proton affinity 39, 40, moreover, the ambient ionization method of MSI enables direct ionization of analytes in raw tissue samples without sample preparation or purification, resulting in severe matrix effects 41, which are responsible for the limited sensitivity for PTX in QMSI analysis and may cause false negative results due to the lack of sensitivity. Compared with other MSI technologies, AFADESI-MSI significantly improves the sensitivity and expands the coverage by introducing high-rate extracting air flow 28. Furthermore, AFADESI-MSI is a spray-based ionization method; thus, the selection of the spray solvent is the most effective and direct approach to enhance AFADESI-MS detection of PTX and PTX-R. The following spray solvents were successively tried to identify the optimal spray solvent system for PTX and PTX-R: ACN/H_2_O (5:5), ACN/H_2_O-0.1% FA (5:5), ACN/H_2_O (8:2), ACN/H_2_O (8:2)-0.1 % FA, ACN/IPA/H_2_O (4:4:2), ACN/IPA/H_2_O (6:2:2), MeOH/H_2_O (5:5), MeOH/H_2_O (5:5)-0.1% FA, MeOH/H_2_O (8:2), MeOH/H_2_O (8:2)-0.1% FA, MeOH/IPA/H_2_O (4:4:2), and MeOH/IPA/H_2_O (6:2:2) (**Figure 2A**). Then, we attempted to add some MS-tolerant volatile salts, such as ammonium formate and ammonium acetate, in the optimal solvent system to further improve the sensitivity of drug detection. As shown in **Figure 2B**, the addition of salt did not improve the sensitivity. Ultimately, the spray solvent system ACN/H_2_O (5:5) was applied for efficient drug and endogenous metabolite desorption, extraction, and ionization to maximize the detection sensitivity and yield accurate quantitative results (**Figure S6**). The quantitative ions selected of PTX and PTX-R under the optimized conditions were [M+Na]^+^ (*m/z* 876.3203) and [M]^+^ (*m/z* 983.4172), respectively **(Figure 2C)**. **Figure 3** illustrates the typical tissue-specific metabolites obtained by AFADESI-MSI under the optimized conditions in highly complicated whole-body animal samples. This high sensitivity, wide coverage AFADESI-MSI technique enables simultaneous visualization of various types of endogenous metabolites, especially highly specific metabolites, which could accurately depict the outline of some organs, such as the heart, liver, lung, brain, spleen, and kidney. Therefore, t-SNE spatial segmentation exhibited good clustering or grouping of different pixels based on metabolite profiling, which enabled the determination of automatically discriminating different physiological regions.

### Spatial-temporal distribution of PTX in whole-body animals

**Figure 4:**
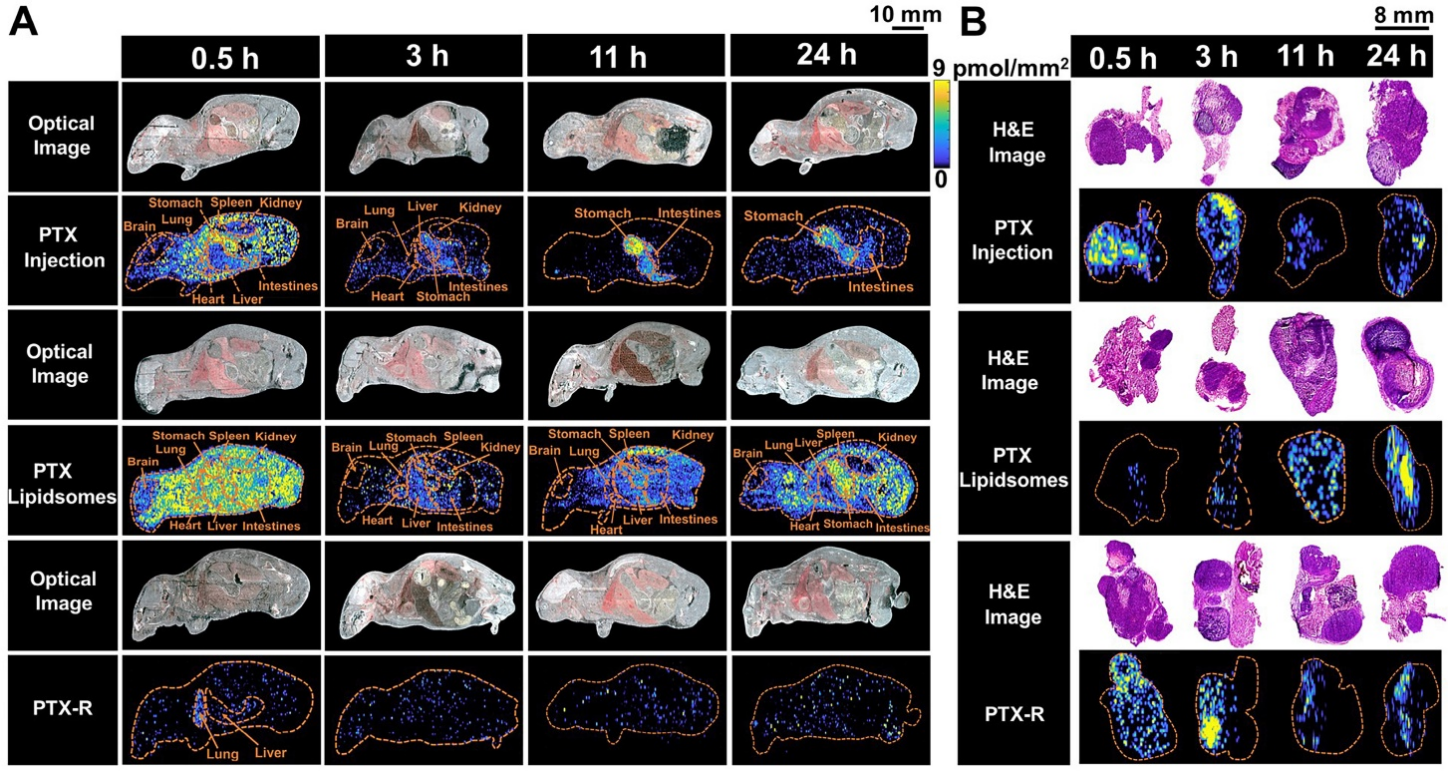
The spatial-temporal and quantitative distribution of PTX in whole-body animals (A) and the corresponding flank tumors (B) at different time points in the three treatment groups visualized by AFADESI-MSI.

**Figure 5:**
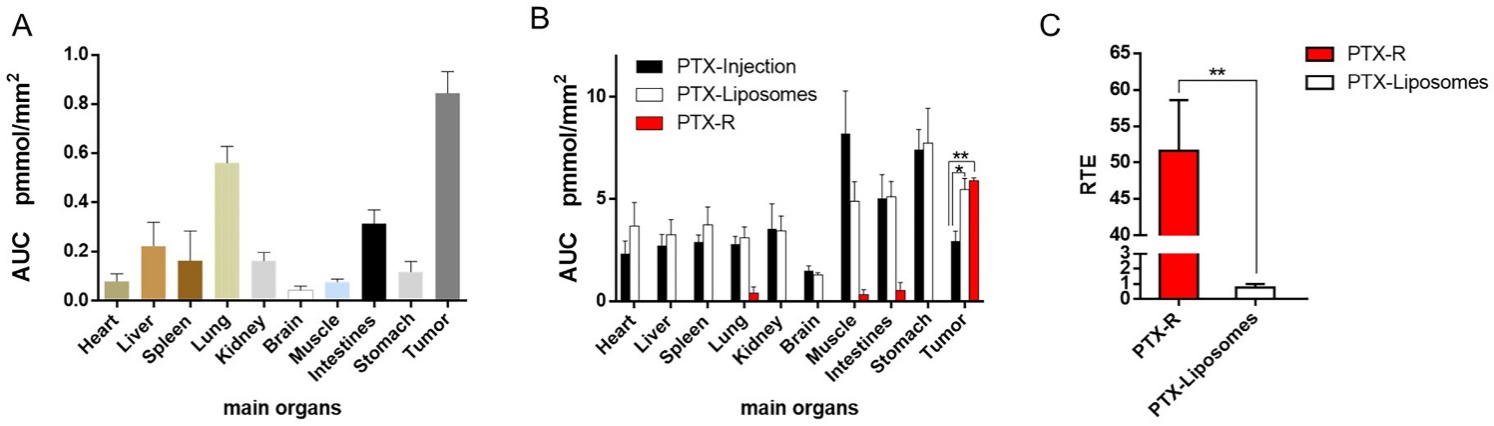
Quantitative statistical analysis of the biodistribution of PTX and PTX-R in whole-body animals analysed by AFADESI-QMSI. (A) Quantification analysis of PTX-R in tumors and major organs within 24 hours after intravenous injection of PTX-R. (B) The absolute quantification results of PTX in different treatment groups within 24 hours. Note: PTX-Injection, xenograft mice treated with PTX injection. PTX-Liposome, xenograft mice treated with PTX liposomes. PTX-R, xenograft mice treated with PTX-R. (C) The relative targeting efficiency of PTX-R and PTX-liposomes relative to PTX injection. The data are presented as the mean±SEM. n=3, *P<0.05, **P<0.01.

To evaluate the tumor-targeting capability of PTX-R, a novel antitumor drug candidate, we carried out VC-QMSI analysis to determine the content per pixel in whole-body animals and flank tumors. Then, the spatial-temporal distribution of PTX and PTX-R was quantitatively visualized in nude mice bearing subcutaneous A549 xenograft tumors treated with three regimens (the PTX-injection group, PTX-liposome group and PTX-R group). PTX was broadly distributed throughout whole-body tissue section of the mice which were treated with PTX-injection and PTX-liposome, and the content in the healthy organs was significantly higher than that of PTX metabolized by the prodrug (PTX-R) from PTX-R-dosed mice (n=3) **(Figure 4A, Figure 5B, Table S4, Figure S7)**. Moreover, a substantially higher PTX accumulation was visualized at the gastrointestinal site than other tissues or organs in PTX-injection and PTX-liposome treated mice, especially at later time points after dosing, and PTX was barely observed in the renal tissues from both groups (**Figure 4A**). These results suggest that PTX is mainly excreted through the bile into the faeces, rather than excreted in the urine through renal, where the main biliary excretion behaviour has also been reported 42. PTX also exhibits drug-related side effects, such as severe diarrhoea, consistent with the drug distribution characteristics detected in the clinic. The parent drug PTX-R also presented similar accumulation and excretion characteristics (**Figure S8A**). A statistical analysis was also performed showing that the prodrug PTX-R significantly accumulated in tumor tissue within 24 hours, followed by the lung and intestine in the group injected with PTX-R (**Figure 5A, Figure S8, Table S3**). As expected, regardless of whether the parent drug or the active drug was examined, no notable drug distribution was observed in the brains of different treatment groups, and this finding might be due to blood brain barrier (BBB) limitations imposed by both the molecular weight and polarity of the drug (**Figure 4A, Figure S8A**).

In addition, we investigated PTX exposure in tumors and compared the three treatment groups (**Figure 4B)**. The area under the concentration-time curve (AUC) in the tumor (AUC_tumor_) for PTX derived from PTX-R was 2-fold higher than that of the PTX-injection group and similarly high with that of the PTX-liposome group. However, the quantity of PTX derived from PTX-R showed extremely low or even no nonspecific accumulation in healthy organs or tissues, indicating low systematic toxicity** (Figure 5B)**. Using PTX injection as a reference, we calculated the relative targeting efficiency (RTE) of PTX-R and PTX-liposomes according to the following formula:


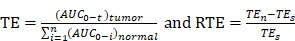


TE_n_ represents the targeting efficiency (TE) of the tested drug, and TE_s_ represents the TE of the reference drug. In contrast, the RTE of PTX-R was approximately 50-fold higher than that of PTX-liposomes, confirming the ability of this drug to specifically target the tumor tissue (**Figure 5C**). Interestingly, the distribution of PTX and PTX-R was highly heterogeneous inside the tumor tissues (**Figure 4B, Figure S8B**).

### Intratumor distribution of PTX-R with heterogeneous characteristics

**Figure 6:**
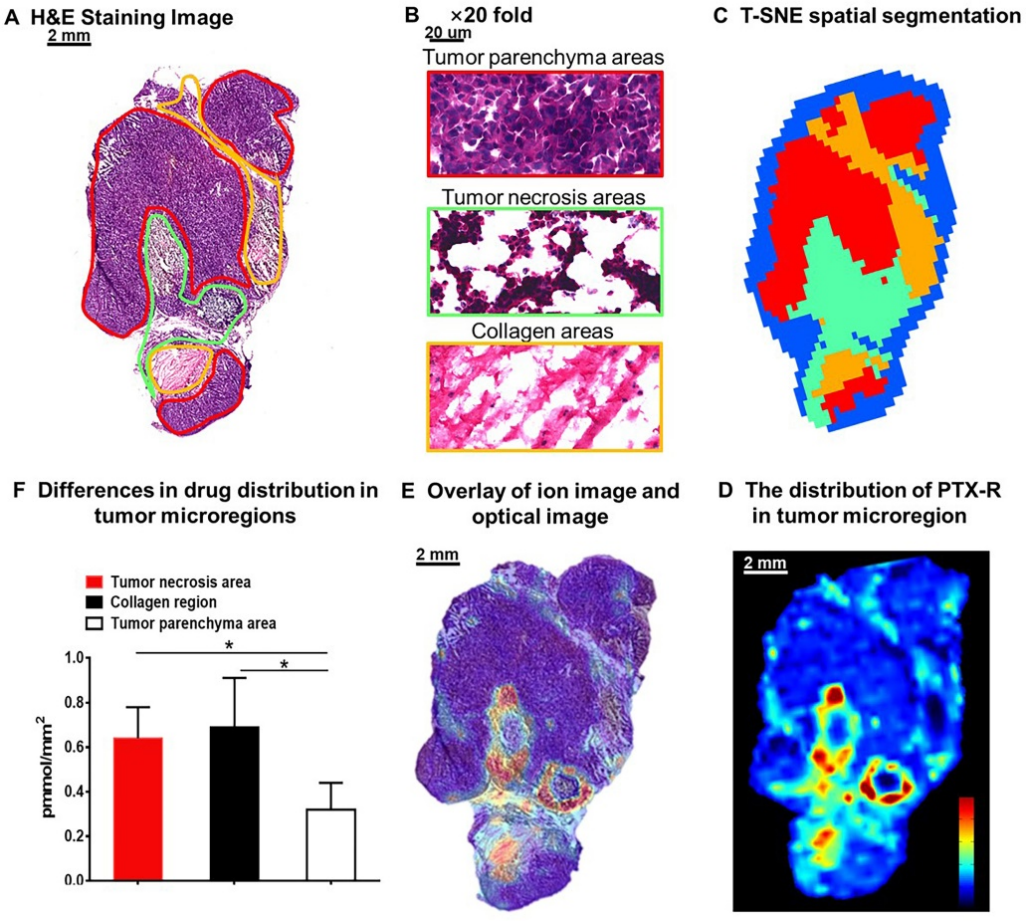
Intratumoral distribution of PTX-R with heterogeneous characteristics. (A) H&E staining image of a tumor tissue. (B) The magnification (×20) figure of each representative tumor microregion. (C) The t-SNE spatial segmentation of the tumor microregion based on metabolite profiling. (D) The distribution of PTX-R in the tumor microregion. (E) The coupling-matching overlay between drug ion imaging and H&E stain imaging. (F) Quantification analysis of PTX-R distribution in the tumor microregion.

The heterogeneous nature of tumor tissue is one of the characteristics of malignant tumors, including intratumor phenotypic or morphological diversity and heterogeneity for drug distribution or sensitivity 43, 44. As shown in **Figure 6A, 6B,** the histopathological assessment results indicated considerable heterogeneity in tumor morphology; all of its constituents, such as the tumor parenchyma cell area (red frame), connective tissue stroma area (orange frame), and vasculature 45, are important parts in the formation of a firm solid tumor framework. According to the cell morphology, the differentiation of the tumor necrosis area can be determined due to the notable cellular debris (green frame). Tumor microregion automatic recognition was based on the metabolomics characteristics of the tumor using t-SNE and k-means and exhibited results that were highly consistent with the H&E staining results (**Figure 6C**). Notably, intratumor differences in the PTX-R levels are visualized in **Figure 6D, 6E**. The accumulation of PTX-R in the tumor necrosis area and collagen region was substantially higher than that in the tumor parenchyma area (**Figure 6F**), indicating that PTX-R has an excellent tumor penetration ability. Another investigation has shown that PTX-R displayed significant accumulation in the poorly differentiated area of the tumor compared with the tumor parenchyma area and tumor stroma area, such as the collagen region and adipose tissue region (**Figure S9**). This heterogeneity of drug distribution in tumor microregions deserves further research and may lead to major differences in tumor growth, invasive ability, sensitivity to drugs, and prognosis.

## Conclusion

In this study, we successfully evaluated the tumor-targeting efficiency and intratumoral heterogeneity of a novel antitumor drug candidate using a label-free and SILIS-free VC-QMSI method, and provided intuitionistic experimental evidence for its excellent tumor-specific accumulation ability and low systemic toxicity. The AFADESI-MSI platform was also shown to be a high sensitivity, wide coverage technique that enables the simultaneous visualization of various types of drugs, metabolites and endogenous metabolites at the whole-body level and in microregions. Then, the abundant endogenous metabolites that are highly correlated with drug MS responses were screened as the “Natural Internal Standards” to build a predictable ANN model. The predicted values of RCF could correct the relative intensities of the drug ion pixel by pixel to achieve quantitative visualization of the drugs in complicated whole-body animal and suborgan tissue samples. The work presented here showed that the quantitative analysis of the distribution of a prodrug (PTX-R) and its active metabolite (PTX) at the whole-body and microregion level could contribute to effective tumor-targeting drug design according to four key considerations: retain, evade, target and release 43. This spatially resolved QMSI analysis also identified the intratumor distribution of PTX-R with its heterogeneous characteristics, which could be related to tumor therapy resistance driven by genetic and epigenetic factors.

The proposed approach will contribute to the prediction of drug efficacy and safety often associated with drug distribution and may enable novel therapeutic screening in a rapid and direct manner at the early stage of antitumor drug research and development, decreasing attrition rates and cost. The study described here also provides deeper insights into the mechanism of drug efficacy, toxicity, and resistance, which will facilitate the design and optimization of antitumor drugs during the drug discovery process.

## Supplementary Material

Supplementary figures and tables, MATLAB source code.

## Abbreviations

PTX: Paclitaxel
PTX-R: Paclitaxel Prodrug
VC-QMSI: Virtual Calibration Quantitative Mass Spectrometry Imaging
WBA: Whole-Body Autoradiography
AFADESI: Airflow-Assisted Desorption Electrospray Ionization
LC-MS: Liquid Chromatography-Mass Spectrometry
t-SNE: t-distributed Stochastic Neighbour Embedding
TE: Targeting Efficiency
RTE: Relative Targeting Efficiency
RCF: Relative Calibration Factors
ANN: Artificial Neural Network
SILIS: Stable-Isotope-Labelled Internal Standard
DMPK: Drug Metabolism and Pharmacokinetics
DDS: Drug Delivery System

## References

[1] Bray F, Ferlay J, Soerjomataram I, Siegel RL, Torre LA, Jemal A (2018). Global cancer statistics 2018: GLOBOCAN estimates of incidence and mortality worldwide for 36 cancers in 185 countries. CA Cancer J Clin.

[2] Vanneman M, Dranoff G (2012). Combining immunotherapy and targeted therapies in cancer treatment. Nat Rev Cancer.

[3] Liang Q, Kong L, Du Y, Zhu X, Tian J (2019). Antitumorigenic and antiangiogenic efficacy of apatinib in liver cancer evaluated by multimodality molecular imaging. Exp Mol Med.

[4] Randall EC, Emdal KB, Laramy JK, Kim M, Roos A, Calligaris D (2018). Integrated mapping of pharmacokinetics and pharmacodynamics in a patient-derived xenograft model of glioblastoma. Nat Commun.

[5] Chen D, Lin X, Zhang C, Liu Z, Chen Z, Li Z (2018). Dual PI3K/mTOR inhibitor BEZ235 as a promising therapeutic strategy against paclitaxel-resistant gastric cancer via targeting PI3K/Akt/mTOR pathway. Cell Death Dis.

[6] Li X, Jeong K, Lee Y, Guo T, Lee D, Park J (2019). Water-Soluble Phthalocyanines Selectively Bind to Albumin Dimers: A Green Approach Toward Enhancing Tumor-Targeted Photodynamic Therapy. Theranostics.

[7] Schrama D, Reisfeld RA, Becker JC (2006). Antibody targeted drugs as cancer therapeutics. Nat Rev Drug Discov.

[8] Angela Casini AS, Claudiu T (2002). Supuran. Cysteine-Modifying Agents: A Possible Approach for Effective Anticancer and Antiviral Drugs. Environmental Health Perspectives.

[9] Mohapatra S, Mallick SK, Maiti TK, Ghosh SK, Pramanik P (2007). Synthesis of highly stable folic acid conjugated magnetite nanoparticles for targeting cancer cells. Nanotechnology.

[10] Li F, Lu J, Liu J, Liang C, Wang M, Wang L (2017). A water-soluble nucleolin aptamer-paclitaxel conjugate for tumor-specific targeting in ovarian cancer. Nat Commun.

[11] Qu X, Qiu P, Zhu Y, Yang M, Mao C (2017). Guiding nanomaterials to tumors for breast cancer precision medicine: from tumor-targeting small-molecule discovery to targeted nanodrug delivery. NPG Asia Mater.

[12] Yin T, Zhang Q, Wu H, Gao G, Shapter JG, Shen Y (2017). *In vivo* high-efficiency targeted photodynamic therapy of ultra-small Fe3O4@polymer-NPO/PEG-Glc@Ce6 nanoprobes based on small size effect. NPG Asia Mater.

[13] Nilsson A, Goodwin RJA, Shariatgorji M, Vallianatou T, Webborn PJH, Andrén PE (2015). Mass Spectrometry Imaging in Drug Development. Anal Chem.

[14] Sun C, Li T, Song X, Huang L, Zang Q, Xu J (2019). Spatially resolved metabolomics to discover tumor-associated metabolic alterations. Proc Natl Acad Sci U S A.

[15] Easwaran H, Tsai HC, Baylin SB (2014). Cancer epigenetics: tumor heterogeneity, plasticity of stem-like states, and drug resistance. Mol Cell.

[16] Reiter JG, Baretti M, Gerold JM, Makohon-Moore AP, Daud A, Iacobuzio-Donahue CA (2019). An analysis of genetic heterogeneity in untreated cancers. Nat Rev Cancer.

[17] Liu Y, Zheng X, Yu Q, Wang H, Tan F, Zhu Q, Yu L, Zeng S (2016). Epigenetic activation of the drug transporter OCT2 sensitizes renal cell carcinoma to oxaliplatin. Sci Transl Med.

[18] Tan T, Hu H, Wang H, Li J, Wang Z, Wang J (2019). Bioinspired lipoproteins-mediated photothermia remodels tumor stroma to improve cancer cell accessibility of second nanoparticles. Nat Commun.

[19] Tellez-Gabriel M, Heymann MF, Heymann D (2019). Circulating Tumor Cells as a Tool for Assessing Tumor Heterogeneity. Theranostics.

[20] Flashner-Abramson E, Vasudevan S, Adejumobi IA, Sonnenblick A, Kravchenko-Balasha N (2019). Decoding cancer heterogeneity: studying patient-specific signaling signatures towards personalized cancer therapy. Theranostics.

[21] Wong AL, Xiang X, Ong PS, Mitchell EQY, Syn N, Wee I (2018). A Review on Liquid Chromatography-Tandem Mass Spectrometry Methods for Rapid Quantification of Oncology Drugs. Pharmaceutics.

[22] Stephen Castellino MRGDW (2011). MALDI imaging mass spectrometry: bridging biology and chemistry in drug development. Bioanalysis.

[23] Matthews PM (2019). Chronic inflammation in multiple sclerosis - seeing what was always there. Nat Rev Neurol.

[24] Li R, Zheng K, Hu P, Chen Z, Zhou S, Chen J (2014). A novel tumor targeting drug carrier for optical imaging and therapy. Theranostics.

[25] Li C, Yang XQ, Zhang MZ, Song YY, Cheng K, An J (2018). *In vivo* Imaging-Guided Nanoplatform for Tumor Targeting Delivery and Combined Chemo-, Gene- and Photothermal Therapy. Theranostics.

[26] Rubakhin SS, Jurchen JC, Monroe EB, Sweedler JV (2005). Imaging mass spectrometry: fundamentals and applications to drug discovery. Drug Discovery Today.

[27] Shariatgorji M, Svenningsson P, Andren PE (2014). Mass spectrometry imaging, an emerging technology in neuropsychopharmacology. Neuropsychopharmacology.

[28] He J, Sun C, Li T, Luo Z, Huang L, Song X (2018). A Sensitive and Wide Coverage Ambient Mass Spectrometry Imaging Method for Functional Metabolites Based Molecular Histology. Adv Sci.

[29] He J, Luo Z, Huang L, He J, Chen Y, Rong X (2015). Ambient mass spectrometry imaging metabolomics method provides novel insights into the action mechanism of drug candidates. Anal Chem.

[30] Luo Z, He J, Chen Y, He J, Gong T, Tang F (2013). Air flow-assisted ionization imaging mass spectrometry method for easy whole-body molecular imaging under ambient conditions. Anal Chem.

[31] Chumbley CW, Reyzer ML, Allen JL, Marriner GA, Via LE, Barry CE 3rd (2016). Absolute Quantitative MALDI Imaging Mass Spectrometry: A Case of Rifampicin in Liver Tissues. Anal Chem.

[32] Sheerin Khatib-Shahidi MA, Jennifer L (2006). Herman, Todd A. Gillespie, and Richard M. Caprioli. Direct Molecular Analysis of Whole-Body Animal Tissue Sections by Imaging MALDI Mass Spectrometry. Anal Chem.

[33] Schulz S, Becker M, Groseclose MR, Schadt S, Hopf C (2019). Advanced MALDI mass spectrometry imaging in pharmaceutical research and drug development. Curr Opin Biotechnol.

[34] Tang W, Chen J, Zhou J, Ge J, Zhang Y, Li P (2019). Quantitative MALDI Imaging of Spatial Distributions and Dynamic Changes of Tetrandrine in Multiple Organs of Rats. Theranostics.

[35] Song X, He J, Pang X, Zhang J, Sun C, Huang L (2019). Virtual Calibration Quantitative Mass Spectrometry Imaging for Accurately Mapping Analytes across Heterogenous Biotissue. Anal Chem.

[36] National Research Council (US) Committee for the Update of the Guide for the Care and Use of Laboratory Animals (2011). Guide for the care and use of laboratory animals, 8nd.

[37] He J, Tang F, Luo Z, Chen Y, Xu J, Zhang R (2011). Air flow assisted ionization for remote sampling of ambient mass spectrometry and its application. Rapid Commun Mass Spectrom.

[38] Lv Y, Li T, Guo C, Sun C, Tang F, Huang L (2019). A high-performance bio-tissue imaging method using air flow-assisted desorption electrospray ionization coupled with a high-resolution mass spectrometer. Chin Chem Lett.

[39] Falcetta F, Morosi L, Ubezio P, Giordano S, Decio A, Giavazzi R (2018). Past-in-the-Future. Peak detection improves targeted mass spectrometry imaging. Anal Chim Acta.

[40] Song X, Luo Z, Li X, Li T, Wang Z, Sun C (2017). *In Situ* Hydrogel Conditioning of Tissue Samples to Enhance the Drug's Sensitivity in Ambient Mass Spectrometry Imaging. Anal Chem.

[41] Zhang W, Wang X, Xia Y, Ouyang Z (2017). Ambient Ionization and Miniature Mass Spectrometry Systems for Disease Diagnosis and Therapeutic Monitoring. Theranostics.

[42] Alex Spareboom OvT, Willem J Nooijen, Jos H Beijnen (1996). Tissue distribution, metabolism and excretion of paclitaxel in mice. Anti-cancer drugs.

[43] Bae YH, Park K (2011). Targeted drug delivery to tumors: myths, reality and possibility. J Control Release.

[44] Zhang J, Fujimoto J, Zhang J, Wedge DC, Song X, Zhang J (2014). Intratumor heterogeneity in localized lung adenocarcinomas delineated by multiregion sequencing. Science.

[45] Bae YH (2009). Drug targeting and tumor heterogeneity. J Control Release.

