# Supplementary material for: Evaluation of the tumor-targeting efficiency and intratumor heterogeneity of anticancer drugs using quantitative mass spectrometry imaging
Source: Theranostics. 2020 Feb 3;10(6):2621–30. doi: 10.7150/thno.41763 (PMC7052894; doi:10.7150/thno.41763)
Supplement: Supplementary file 1 — Supplementary figures and tables, MATLAB source code. [file thnov10p2621s1.pdf]

Supporting information

Evaluation of the tumor-targeting efficiency and intratumor heterogeneity of anticancer drugs using quantitative mass spectrometry imaging

Jin Zhang[1], Qianqian Du[2], Xiaowei Song[1], Shanshan Gao[1], Xuechao Pang[1], Yan Li[2], Ruiping Zhang[1], Zeper Abliz[1,3], Jiuming He[1]✉

1 State Key Laboratory of Bioactive Substance and Function of Natural Medicines, Institute of Materia Medica, Chinese Academy of Medical Sciences and Peking Union Medical College, Beijing, 100050, China

2 Beijing Key Laboratory of New Drug Mechanisms and Pharmacological Evaluation Study, Institute of Materia Medica, Chinese Academy of Medical Sciences and Peking Union Medical College, Beijing, 100050, China

3 Center for Imaging and Systems Biology, Minzu University of China, Beijing, 100081, China

✉Corresponding author: Tel/Fax: +010-63165218. Email address: hejiuming@imm.ac.cn (J.M. He)

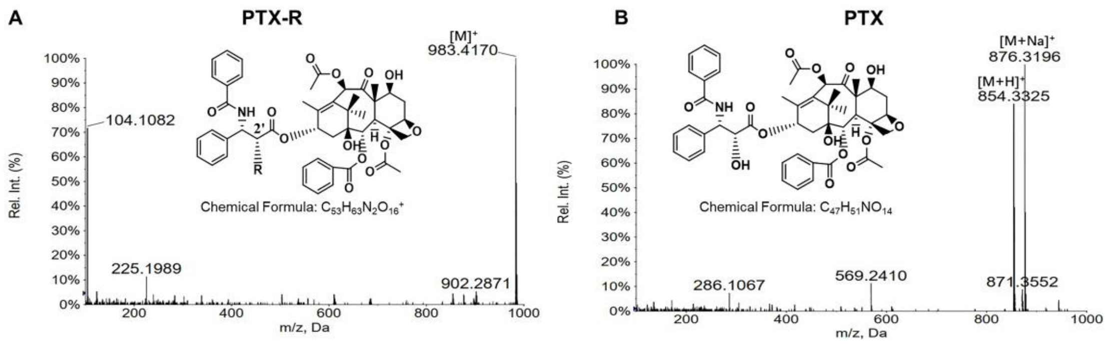

Figure S1. The chemical structure and high-resolution mass spectrum of PTX-R (A) and PTX (B).

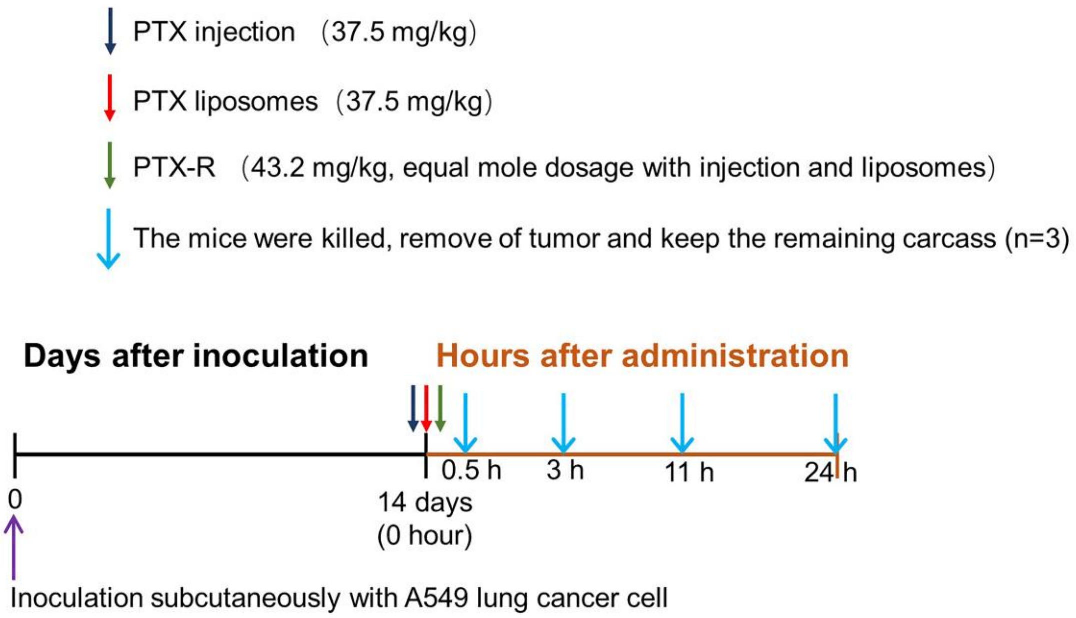

Figure S2. Detailed schedule for the treatment scheme in the xenograft tumor model

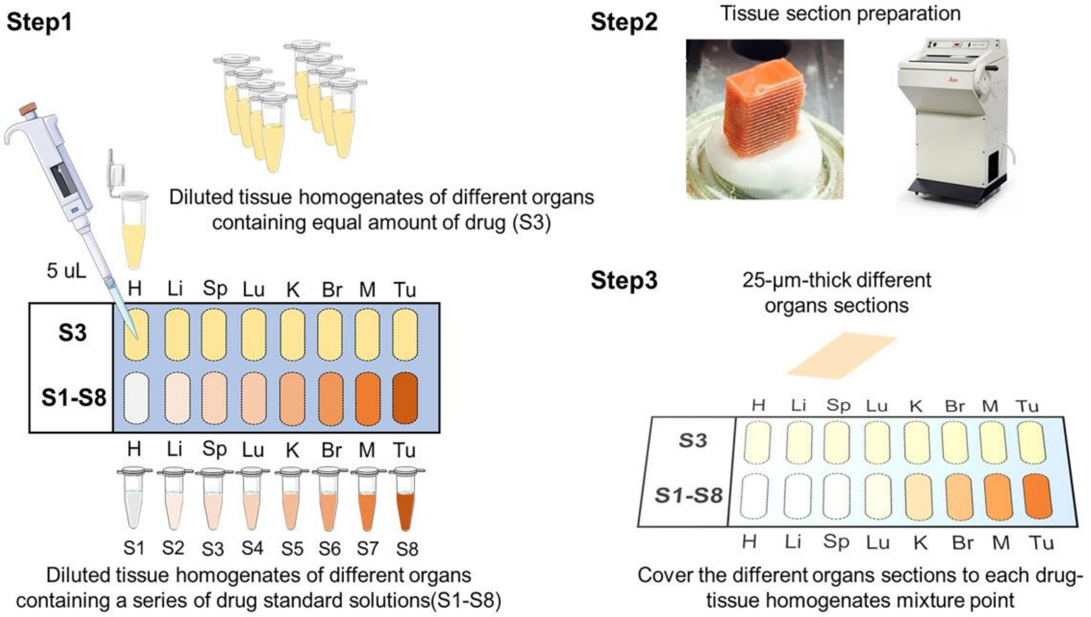

Figure S3. Schematic illustration of mimetic tissue models preparation.

Step1: 5 μL of the diluted tissue homogenates of different organs (H, heart; Li, liver; Sp, spleen; Lu, lung; K, kidney; Br, brain; M, muscle; Tu, tumor;) containing equal amount of drug (S3, 4.45 pmol/mm^2 for PTX and 0.79 pmol/mm^2 for PTX-R) was drawn into the well of a self-custom mold; 5 μL of the diluted tissue homogenates of different organs (H, Li, Sp, Lu, K, Br, M, Tu) containing a series of drug standard solutions (S1-S8, 0.89, 1.78, 4.45, 8.9, 13.35, 17.8, 35.6, and 71.2 pmol/mm^2 for PTX and 0.079, 0.158, 0.79, 1.58, 3.95, 7.9, 15.8, and 31.6 pmol/mm^2 for PTX-R) were drawn into the well of a self-custom mold and a holding time of 5 mins was used to allow the sample to dry.

Step 2: Tissue section preparation of different organs.

Step 3: Each dried point was covered using the tissue sections of the corresponding organ.

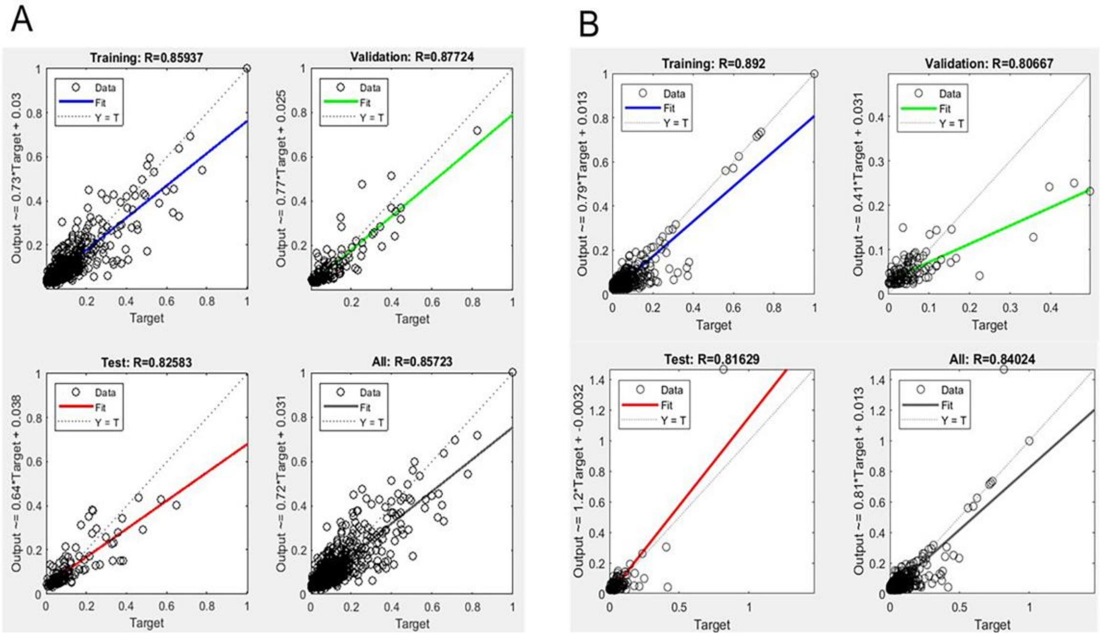

Figure S4. The neutral network training result of predicting the relative calibration factors of PTX-R (A) and PTX (B) using endogenous metabolites

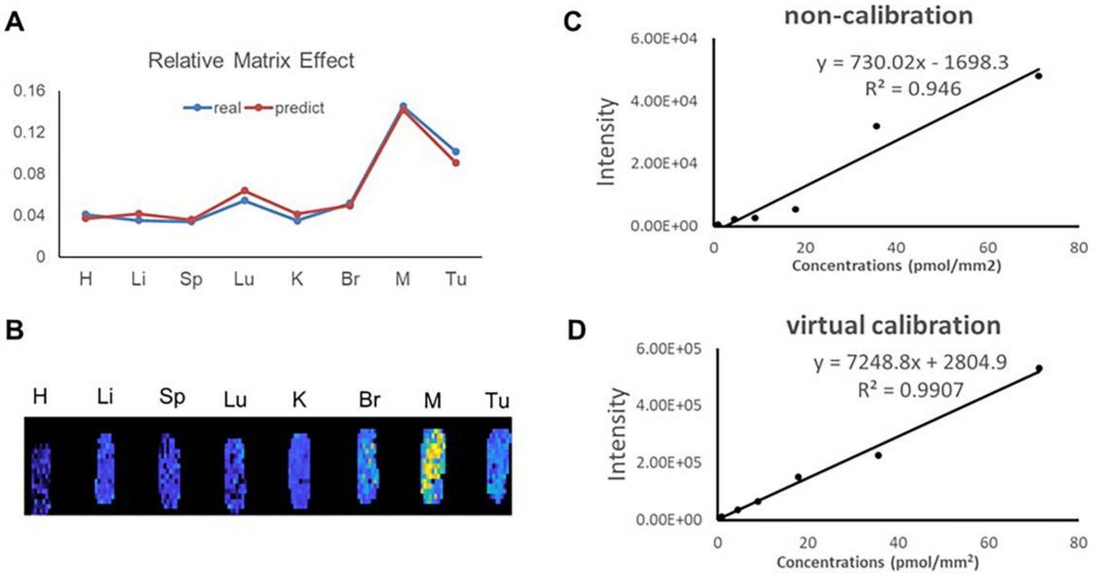

Figure S5. The VC-QMSI strategy modeling results for PTX. (A) The comparison of predicted and true values of the relative calibration factor. (B) Imaging visualization of the predicted relative calibration factor. (C) and (D) The non-calibration and virtual calibration standard curve constructed with the drug amount versus non-calibrated and calibrated PTX intensities, respectively.

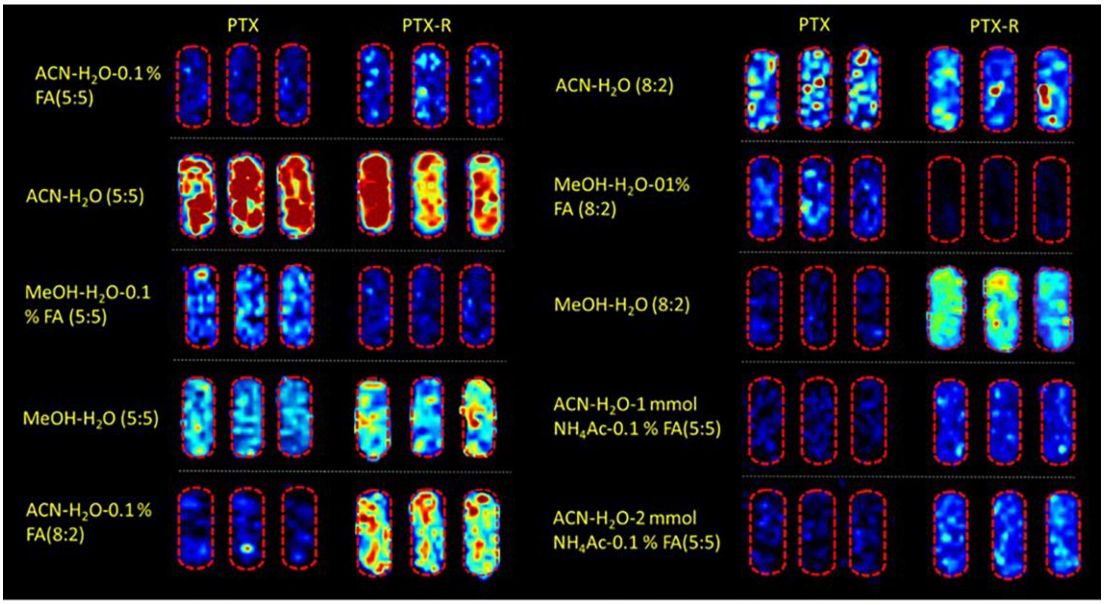

Figure S6. Representative MS images of PTX and PTX-R in equivalent amount drug-spiked mimetic tissue models under different composition of the spray solvent.

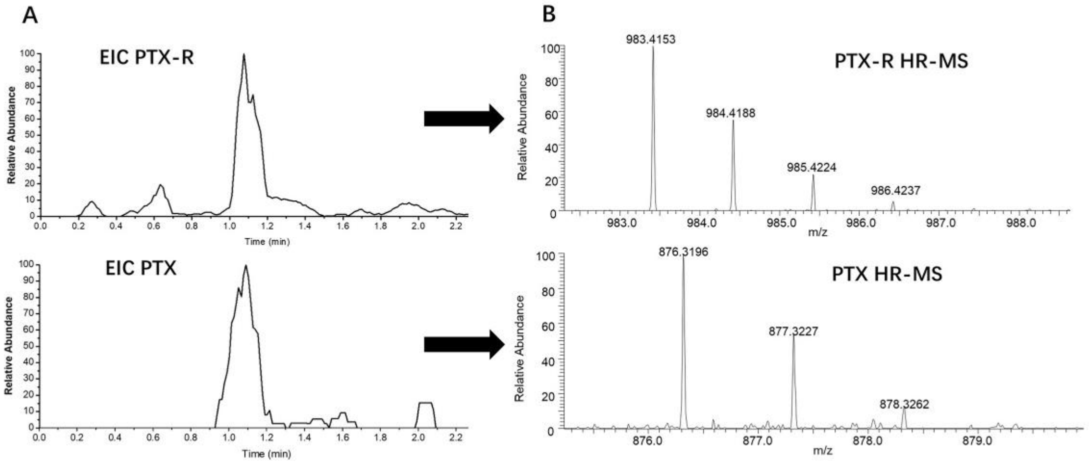

Figure S7. The extracted ion chromatograms of prodrug (PTX-R) and metabolized paclitaxel (PTX) from the raw MSI data of the nude mouse dosed with PTX-R (A) and corresponding high-resolution mass spectrum (B)

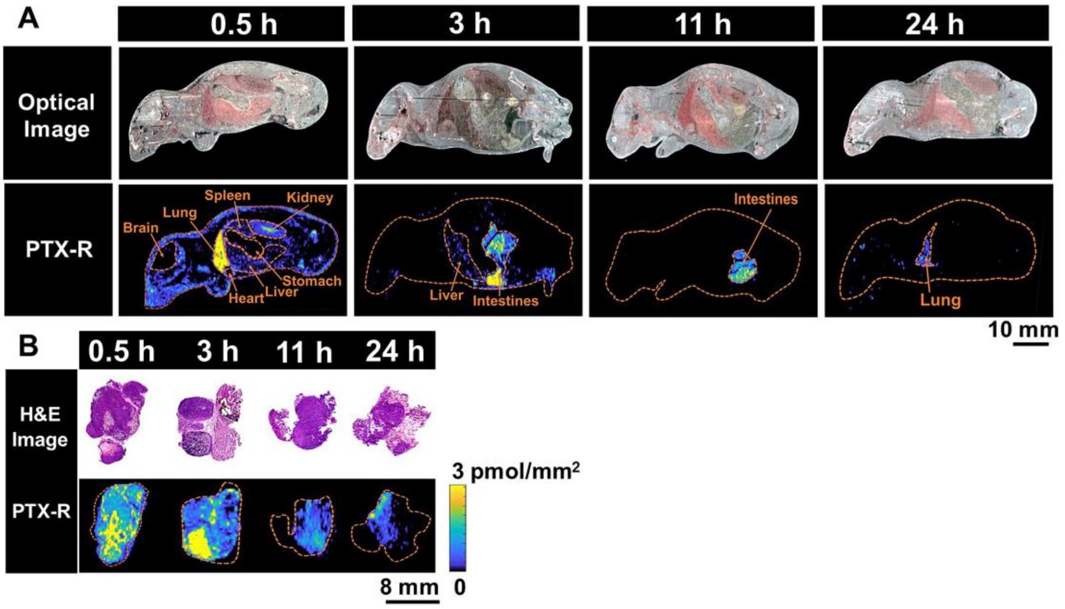

Figure S8. The spatial-temporal and quantitative distribution of PTX-R in whole-body animals (A) and corresponding flank tumors (B) at different time points visualized by AFADESI-MSI.

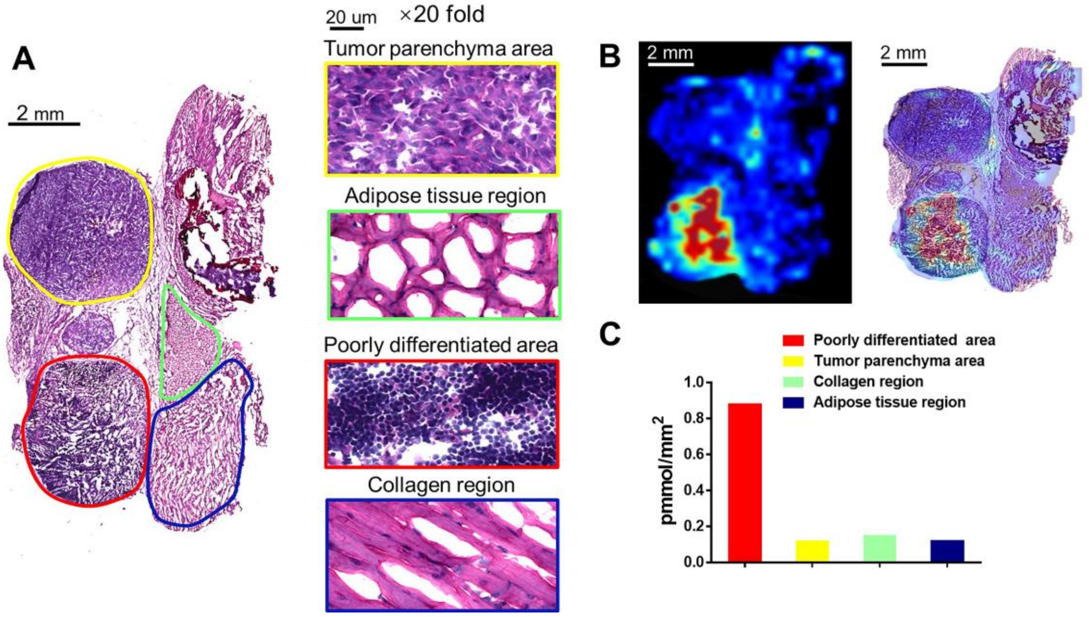

Figure S9. Intratumoral distribution of PTX-R with heterogeneous characteristics. (A) H&E staining image of a tumor tissue and the magnification (×20) figure of each representative tumor microregion. (B) The visualization of PTX-R in tumor microregion and the coupling-matching overlay between PTX-R imaging and H&E stain imaging. (C) The quantification analysis of PTX-R distribution in tumor microregion.

Table S1 Key parameters of AFADESI-MSI platform.

	**Key Parameters**	**Setting Value**
**Ion source**	Spray voltage	7000 V
	Transport Tube voltage	0 V
	Spray gas pressure	0.7 MPa
	Extracting gas flow	45 L/min
	Spray solvent composition	Acetonitrile-water (5:5, v:v)
	Spray solvent flow	10 μL/min
	X axis moving speed	0.35 mm/s
	Y axis step size	0.5 mm
**Q Exactive Mass spectrometer**	Scan mode	Full MS/t-SIM
	Mass range	Full MS: *m/z* 100-1000; t-SIM: *m/z* 830-880(PTX); *m/z* 950-1000 (PTX-R)
	Polarity	Positive
	Maximum inject time	100 ms for Full MS; 300 ms for t-SIM
	AGC target	5e6 for Full MS; 3e6 for t-SIM
	Resolution	70000
	Capillary temperature	350 ℃

Table S2 The screened endogenous metabolites as native internal standards for quantifying PTX-R and PTX in VC-QMSI, respectively.

	Endogenous metabolites (*m/z*)	
	PTX	PTX-R
1	112.0870	112.0870
2	437.1961	568.3444
3	438.1994	874.7818
4	569.2410	985.4382
5	605.4011	986.4415
6	692.4197	/
7	693.3649	/

Table S3 The summary of p-value differences in PTX-R distribution between any two organs.

	Heart	Liver	Spleen	Lung	Kidney	Brain	Muscle	Intestines	Stomach	Tumor
Heart	/	0.23	0.54	**0.0030**	0.15	0.36	0.94	**0.020**	0.50	**0.0012**
Liver	0.23	/	0.72	**0.046**	0.58	0.14	0.21	0.46	0.37	**0.0012**
Spleen	0.54	0.72	/	**0.046**	0.99	0.38	0.52	0.32	0.74	**0.010**
Lung	0.0030	0.046	0.046	/	0.0065	0.0018	0.0022	**0.048**	0.0052	0.063
Kidney	0.15	0.58	0.99	**0.0065**	/	0.039	0.083	0.079	0.46	**0.0020**
Brain	0.36	0.14	0.38	**0.0018**	0.039	/	0.17	**0.0092**	0.18	**0.00088**
Muscle	0.94	0.21	0.52	**0.0022**	0.083	0.17	/	**0.013**	0.40	**0.00099**
Intestines	0.020	0.46	0.32	**0.048**	0.079	0.0092	0.013	/	0.046	**0.0070**
Stomach	0.50	0.37	0.74	**0.0052**	0.46	0.18	0.40	**0.046**	/	**0.0017**
Tumor	0.0012	0.0012	0.010	0.063	0.0020	0.00088	0.00099	**0.0070**	0.0017	/

$p<0.05$

Table S4 The summary of p values for differences in PTX distribution between any two treatment groups.

	PTX-R/Lipidsomes	PTX-R/Injection	Injection/Lipidsomes
Heart	0.058	**0.040**	0.44
Liver	**0.022**	**0.014**	0.66
Spleen	**0.024**	**0.0023**	0.51
Lung	**0.015**	**0.0091**	0.71
Kidney	**0.017**	**0.046**	0.84
Brain	**0.00040**	**0.0071**	0.57
Muscle	**0.018**	**0.018**	0.30
Intestines	**0.0091**	**0.0091**	0.97
Stomach	**0.020**	**0.020**	0.90
Tumor	0.56	**0.0085**	**0.044**

$p<0.05$

MATLAB source code for data processing in VC-QMSI of PTX and PTX-R [1]

Steps of VC-QMSI

(1) Data preprocessing
(2) Regression modeling
(3) Quantitative standard curve establishment
(4) Spatial segmentation
(5) Virtual calibration and quantitation

Self-written MATLAB Scripts

(1) Data preprocessing: peaklist and datacube; indexing; dimension reduction
(2) Spatial segmentation
(3) Regression modelling
(4) Virtual Calibration and QMSI

Self-defined MATLAB Functions

(1) peaklist=batchmzxmlread(); %read batch of mzXML files and save into peaklist
(2) peaklist=batchcdfread('file_prefix',file_numbers); % read batch of cdf files and save into peaklist
(3) drug=selectimagechanel(drug,channel_no); % select the odd or even channel to form a separate image
(4) rangenorm() % make certain variable's value range in [0, 1] interval
(5) rsd() % calculate the relative standard deviation
(6) loge() % make natural logarithm transformation
(7) indximg(vector, row_index, column_index) % to construct the ion image from a vector composed of pixel values according to their row and column index.
(8) massimage(peaklist, target_mz, mass_tolerance) % to construct the ion image by extracting the target m/z within each mass spectrum in each file.
(9) [datacube] = batchmassimage(peaklist,cmz,mass_tolerance) % to construct serial images of ions from cmz, the element was extracted from the correspondent certain file (row) and certain scan (column) in peaklist within the self-defined mass tolerance.

Variables used in the scripts

peaklist % the cell contains all of scan files
datacube % the set of images constructed with series of ions in mass spectrum
biocube % the set of bio-informative images
biomat % the 2D matrix only contains those pixels within the tissue region.
cmz % the vector composed of a column of target metabolite ions and target ion
indx_pk % the index of all peaks of interest
indx_pix % the index of pixels. It contains three column representing the unique ID No, row index and column index, respectively.
indx_biopix % the index of pixels which belong to the biological sample.
indx_biopk % the index of peaks which belong to endogenous metabolites.
indx_vcpk % the index of analyte response-related peaks used for virtual calibration.
train_mat % the final matrix used for training machine learning model.

target_vector % the target for training dataset.
input % the final matrix put into the machine learning-based regression model.
output % the result based on model prediction.
label % the spatial segmentation result for assigning a pixel to certain organ or tissue.
label_image % the 2D image of the spatial segmentation results.
tsne_feature % the general tSNE features extracted from the screened region-specific metabolite ions.
rcf1D % the vector composed of predicted relative calibration factors.
rcf2D % the matrix of predicted pixel's relative calibration factor based on its location.
drug2D % the drug ion image.
drug_vc % the virtually calibrated ion intensity of drug.
drug_quant % the vector composed of drug quantity in each pixel calculated with QMSI standard curve.
wb_quan_subset % the cell composed of quantitative drug distribution result in each organ or tissue region.
wb_region_subset % the cell composed of several organ subsets, which composed of its regional pixel index.
drug_stata % the statistic information of raw drug ion intensity.
drug_vc_stata % the statistic information of the virtually calibrated drug ion intensity.
drug_quant_stata % the statistic information of the quantified drug amount.

Step (1) Data preprocessing

```
% reading series of No *.cdf files
% saved all of raw data into a cell named peaklist
peaklist=batchmzcdfread('file_prefix',number_of_files);
% extract the image of drug ion ([M]+, m/z 983.4172) with the mass tolerance±0.005
% Suppose the whole-body sample was scanned under alternative scan mode (Full MS/t-SIM).
% then there will be two channels as follows:
% Channel 1 is Full MS for searching drug response-related ions (m/z 100-1000)
% Channel 2 is t-SIM for detecting drug ions
drug=massimage(peaklist,983.4172,0.005);
drug=selectimagechanel(drug,2);
% Visualization of Drug distribution across sections
% ROI selection
% manually select the pixels within ROI region (Sd1-Sd8)
% saved them into a cell named roi_sd
imagesc(drug);
roi_sd{1,1}=roipoly;
roi_sd{2,1}=roipoly;
roi_sd{3,1}=roipoly;
roi_sd{4,1}=roipoly;
roi_sd{5,1}=roipoly;
roi_sd{6,1}=roipoly;
roi_sd{7,1}=roipoly;
```

```
roi_sd{8,1}=roipoly;
roi_drug=roi_sd{1,1}+roi_sd{2,1}+roi_sd{3,1}+roi_sd{4,1}+roi_sd{5,1}+roi_sd{6,1}+roi_sd{7,
1}+roi_sd{8,1};
% m/z values of endogenous metabolites ions were saved in cmz vector
% construte sets of ion images based on the peaks in cmz vector
% cmz vector is composed of main peaks (intensity>1E4) in the average spectrum in whole-body
sections
datacube=batchmassimage(peaklist,cmz,0.005);
for i=1:length(cmz)
        datacube{i,2}=selectimagechanel(datacube{i,2},1);
        if size(datacube{i,2},2)~=size(drug,2)
                datacube{i,2}=datacube{i,2}(:,1:end-1);
        end
end
clear i
clc
% Peak Selection
% Selection rule: peaks with S/N > =2 (self-determined threshold)
% S/N of certain ion was defined as the ratio of average intensities across sample region versus
that within background region.
% If S/N of certain ion is smaller than the setted threshold value, that
% ion will be considered as the background ion and ruled out.
% The remained ions in cmz vector will be saved for the following process
sn_threshold=2;
n=0;
for i=1:length(datacube)
      temp=datacube{i,2};
      x=temp(find(roi_drug==1));
      y=temp(find(roi_drug==0));
      if mean(x)>=sn_threshold*mean(y);
              n=n+1;
              biocube{n,1}=datacube{i,1};
              biocube{n,2}=datacube{i,2};
      end
      temp=[];
end
clear i n temp x y sn_threshold
clc
% Pixel and Peak registration
% edit the index information for each pixel and save it as pix_indx(row_indx, col_indx)
% edit the index information for each peak and save it as pk_indx(Peak No, peak value)
rows=size(drug,1);columns=size(drug,2);
col_indx=repmat([1:columns]',rows,1);
temp=repmat([1:rows],columns,1);
```

```
row_indx=temp(:);
no_indx=1:length(drug(:));
pix_indx=[no_indx',row_indx,col_indx];
clear col_indx row_indx no_indx temp columns rows
pk_indx=[[1:length(cell2mat(biocube(:,1)))]',cell2mat(biocube(:,1))];
clc
% Pick up the data from those pixels within the ROI regions
% followed by constructing the training dataset named biomat.
% In the biomat matrix,each column represents one metabolite ion,each row represents one pixel's
endogenous metabolite ions.
for i=1:length(biocube)
        temp=biocube{i,2}.*roi_drug;
        biomat(:,i)=reshape(temp',size(temp,1)*size(temp,2),1);
end
% Pick out the data from those pixels within the ROI region from drug ion or its isotope ion image
% and construct the target for the training dataset.
key=roi_drug';
biopix_indx=pix_indx(find(key(:)==1),:);
biomat=biomat(find(key(:)==1),:);
drug_temp=drug';
target=drug_temp(find(key(:)==1));
clear i temp key drug_temp
clc
% To check if the biomat was correctly constructed by pixel/peak selection
% column(m/z) vector from biomat was manually selected to reconstruct the ion image
imagesc(indximg(biomat(:,1),biopix_indx(:,2),biopix_indx(:,3)));
figure(2)
imagesc(indximg(target,biopix_indx(:,2),biopix_indx(:,3)));
% Feature Selection
% Features should be selected to form input of training dataset.
% Those metabolite ions which have similar variation with the drug ion
% across different simulative organ sections could be considered as
% drug response related ions or the features.
% Pearson correlation coefficient "r" was employed to screening features.
% Select Top N (2~10) variables from correlation vector as the input feature
% extract the corresponding column data from biomat to form the input training dataset
r_threshold=0.4;
for i=1:size(biomat,2)
        correlation(i,1)=corr(biomat(:,i),target);
end
correlation=[pk_indx,correlation];
feature=correlation(find(correlation(:,3)>=r_threshold),1:3);
train=biomat(:,(feature(:,1)'));
clear i
```

```
clc
% make pre-processing of the input and output training dat
input=rangenorm(train);
output=rangenorm(target);
```

Step (2) Regression modeling

```
% Artifical Neural Network (ANN)
% Solve an Input-Output Fitting problem with a Neural Network
% This script assumes these variables are defined:
%     input - input data.
%     output - target data.
x=rangenorm(train);
y=rangenorm(target);
x=x';
t=y';
% Choose a Training Function
trainFcn = 'trainlm';   % Levenberg-Marquardt
% Create a Fitting Network
hiddenLayerSize = 10;
net = fitnet(hiddenLayerSize,trainFcn);
% Setup Division of Data for Training, Validation, Testing
net. divideParam. trainRatio = 70/100;
net. divideParam. valRatio = 15/100;
net. divideParam. testRatio = 15/100;
% Train the Network
[nn,tr]=train(net,x,t);
% Test the Network
y=net(x);
e=gsubtract(t,y);
performance=perform(net,t,y)
% View the Network
view(net)
%test the fitted model with the real section data
rcf1D'=net.net(xx');
rcf1D=rcf1D';
rcf2D=indximg(rcf1D,indx_biopix(:,2),indx_biopix(:,3));
imagesc(rcf2D);
```

Step (3) Quantitative standard curve establishment

```
% Visualization of drug standard curve
% ROI selection
% manually select the pixels within ROI region (Sd1-Sd8)
% saved them into a cell named roi_sd
imagesc(drug);
```

```
drug_vc= drug./rcf2D;
roi_sd{1,1}=roipoly;
roi_sd{2,1}=roipoly;
roi_sd{3,1}=roipoly;
roi_sd{4,1}=roipoly;
roi_sd{5,1}=roipoly;
roi_sd{6,1}=roipoly;
roi_sd{7,1}=roipoly;
roi_sd{8,1}=roipoly;
roi_drug=roi_sd{1,1}+roi_sd{2,1}+roi_sd{3,1}+roi_sd{4,1}+roi_sd{5,1}+roi_sd{6,1}+roi_sd{7,
1}+roi_sd{8,1};
% to calculate the drug intensity in each organ labeled from 1 to 8
for i=1:length(roi_sd)
    temp=drug_quant(find(roi_sd {i,2}==1));
    temp=temp(find(temp>0));
    drug_stata(i,:)=[mean(temp),std(temp),rsd(temp)];
    temp=[];
end
clear i
clc
```

Step (4) Spatial segmentation

```
% reading batch No *.cdf file and save them into a cell named peaklist
peaklist=batchmzcdfread('file_prefix_name',file_numbers);
% using signature or cmz vector to construct the biocube which is composed
% of correpondent m/z value in the 1st column and its 2D image data in the 2nd column.
mass_tolerance=0.005;
datacube=batchmassimage(peaklist,cmz,mass_tolerance);
for i=1:length(cmz)
    datacube{i,2}=selectimagechanel(datacube{i,2},1);
    if size(datacube{i,2},2)~=size(drug,2)
        datacube{i,2}=datacube{i,2}(:,end-1);
    end
end
clear i
clc
% extract the image of typical high-abundance metabolite ion,choline m/z 104.1071,
% as the reference region of sample
distance=abs(162.1123-cell2mat(datacube(:,1)));
key=find(distance==min(distance));
imagesc(datacube{key,2});
clear distance key;
clc
% manually select the whole-body section region named as roi_bio
roi_bio=roipoly;
```

```
% transfer from 3D biocube into 2D biomatrix
for i=1:length(datacube)
    temp=datacube{i,2}.*roi_bio;
    temp=temp';
    datamat(:,i)=temp(:);
end
key=roi_bio';
biopix_indx=pix_indx(find(key(:)==1),:);
biomat=datamat(find(key(:)==1),:);
clear i temp key
clc
% to validate if the dimension reduction result was correct
imagesc(indximg(biomat(:,1),biopix_indx(:,2),biopix_indx(:,3)));
% Unsupervised pixel clustering
% data standardization
biomat_std=zscore(biomat);
% t-SNE dimension reduction from 50 variables into 3 features
tsne_feature=tsne(biomat_std,[],3,50);
%The class label was defined as No.1-14
%The pixels within sample region was clustered using kmeans clustering
label=kmeans(tsne_feature,10);
%display the clustering results
scatter3(tsne_feature(:,1),tsne_feature(:,2),tsne_feature(:,3),20,label,'fill');
xlabel('tSNE1');ylabel('tSNE2');zlabel('tSNE3');
figure(2)
%recheck the clustering results by visualization of label image
label_image=indximg(label,biopix_indx(:,2),biopix_indx(:,3));
imagesc(label_image);
colormap(jet(15));
```

Step (5) Virtual calibration and quantitation

```
% with virtual calibration, quantified with single standard curve
rcf2D=indximg(pred_rcf,indx_biopix(:,2),indx_biopix(:,3));
drug_vc=drug./rcf2D;
%after calibration, direct quantitation at any point can be calculated with single standard curve
% in this example, the slope and intercept of the virtually calibrated
% standard curve is 89085 and 251437, respectively
drug_quant=(drug_vc-intercept)/slope;
% to calculate the drug quantity in each organ labeled from 1 to 10
for i=1:length(wb_quan_region)
    temp=drug_quant(find(wb_quan_region{i,2}==1));
    temp=temp(find(temp>0));
    drug_stata(i,:)=[mean(temp),std(temp),rsd(temp)];
    temp=[];
```

```
end
clear i
clc
```

References

1. Song X, He J, Pang X, Zhang J, Sun C, Huang L, et al. Virtual Calibration Quantitative Mass Spectrometry Imaging for Accurately Mapping Analytes across Heterogenous Biotissue. Anal Chem. 2019; 91: 2838-2846.
